# Transformer fault identification based on GWO-optimized Dual-channel M-A method

**DOI:** 10.1371/journal.pone.0312474

**Published:** 2024-10-28

**Authors:** Ning Ji, Xi Chen, Xue Qin, Wei Wei, Chenlu Jiang, Yifan Bo, Kai Tao

**Affiliations:** 1 Skill Training Center of State Grid Jiangsu Electric Power Co., Ltd., Suzhou, China; 2 College of Automation & College of Artificial Intelligence, Nanjing University of Posts and Telecommunications, Nanjing, China; Amrita Vishwa Vidyapeetham Amrita School of Engineering Bengaluru, INDIA

## Abstract

In order to improve the accuracy of the transformer fault identification using nature-inspired algorithms, an identification method based on the GWO (Grey Wolf Optimizer)-optimized Dual-channel MLP (Multilayer Perceptron)-Attention is proposed. First, a Dual-channel model is constructed by combining the AM (Attention Mechanism) and MLP. Subsequently, the GWO algorithm is used to optimize the number and the nodes of the hidden layer in the Dual-channel MLP-Attention model. Typical transformer faults are simulated using DDRTS (Digital Dynamic Real-Time Simulator) system. Experiments showed that the GWO- optimized method has an accuracy rate of 95.3%-96.7% in identifying the transformer faults. Compared with BP, SVM, MLP, and single-channel M-A models, the proposed method improved the accuracy by14.1%, 9.6%, 9.3%, and 3.3% respectively. This result indicates the rationality and effectiveness of the proposed method in transformer fault identification.

## 1 Introduction

Transformer is a vital component in power systems [1]. The working environment of transformer is remote and harsh, which is prone to faults, such as insulation aging, short circuit, etc. [2–4] Transformer faults not only affect the operation of power system, but also could lead to significant accidents [5–9]. Therefore, it is of great significant to identify the transformer faults.

Nature-inspired algorithms and artificial intelligence technology have been widely used in the fault identification [10–14], for example, Support Vector Machine [15], Random Forest [16], Multilayer Perceptron [17] as well as Bayesian method [18], etc. Paul et al. [19] researched a gradient boosting (GB) model to optimize the Bayesian parameters. Liao et al. [20] proposed a transformer fault diagnosis model which integrates high accuracy and interpretability. Wang et al. [21] presented a TPE-XGBoost model with a identification accuracy of 89.5% in the condition of 20% missing data.

Nature-inspired algorithms are applicable in the field of power systems. However, there are various types of transformer faults, such as abnormal temperature, partial discharge, etc. Faults would be coupled. When a local fault occurs, it may cause the fluctuations in other parts, leading to the expansion of the accident. This characteristic makes traditional identification models insufficient in capturing the random features and potential fault modes.

The substation recording signal contains key fault information, which could be used for fault identification. A novel transformer fault identification method based on GWO (Grey Wolf Optimizer) and Dual-channel MLP (Multi Layer Perceptron)-Attention was proposed in this paper. Traditional identification methods have poor diagnostic performance for the complex faults. The number of hidden layers and nodes in the MLP-Attention model could be optimized using the GWO algorithm. In this way, the transformer fault could be quickly identified, so that the equipment damage accidents could be prevented. Moreover, this method could assist in analyzing the cause of fault, which is helpful for the stable operation of the system.

## 2 Methodology

### 2.1 GWO

GWO is a nature-inspired optimization algorithm that simulates the hunting behavior of grey wolves [22]. In a gray wolf pack, there is a leading gray wolf (α), several secondary leading gray wolves (β). The rest are ordinary wolves (δ) and the bottom wolves (ω). The alpha wolf represents the current best solution [23]. The process of searching for prey could be described as follow

$$\{\begin{array}{c} D=|C\cdot z_{p}(t)-z(t)|\\ z(t+1)=z_{p}(t)-A\cdot D\\ A=2a(r_{1}-1)\\ C=2r_{2}\\ a=2(1-\frac{t}{t_{max}}) \end{array},$$ (1)

where D is the distance between the individual and the prey, A is the convergence factor, C is the oscillation factor. t is the iteration counts. *z* and *z*_*p*_ are the positions of the grey wolf and the prey respectively. a linearly decreases from 2 to 0. *r*_1_, *r*_2_ ∈ (0,1). During the search and capture of prey, instructions are given by the alpha, beta, as well as the delta wolves. The positions of the top 3 wolves in terms of fitness are preserved during the iterations. The position information of the other wolves is updated,

$$\{\begin{array}{c} D_{\alpha }=|C_{1}\cdot z_{\alpha }-z|\\ D_{\beta }=|C_{2}\cdot z_{\beta }-z|\\ D_{\delta }=|C_{3}\cdot z_{\delta }-z| \end{array},$$ (2)

$$\{\begin{array}{c} z_{1}=z_{\alpha }-A_{1}D_{\alpha }\\ z_{2}=z_{\beta }-A_{2}D_{\beta }\\ z_{3}=z_{\delta }-A_{3}D_{\delta } \end{array},$$ (3)

$$z(t+1)=\frac{z_{1}+z_{2}+z_{3}}{3},$$ (4)

where *D*_*α*_, *D*_*β*_, *D*_*δ*_ represent the distances between the wolves α, β, δ and prey. *A*_1_, *A*_2_, *A*_3_ are coefficient vectors of wolves α, β, δ. *z* is the position of wolf individual. Initialize the population size and the positions. Let *t*_*max*_ be the maximum number of iterations. Compute the fitness based on the initialized positions, so that the positions of wolves α, β, δ, and ω could be calculated. Evaluate whether the maximum number of iterations has been reached, so that the optimal number of hidden layers and hidden layer nodes could be obtained.

### 2.2 MLP

**Fig 1:**
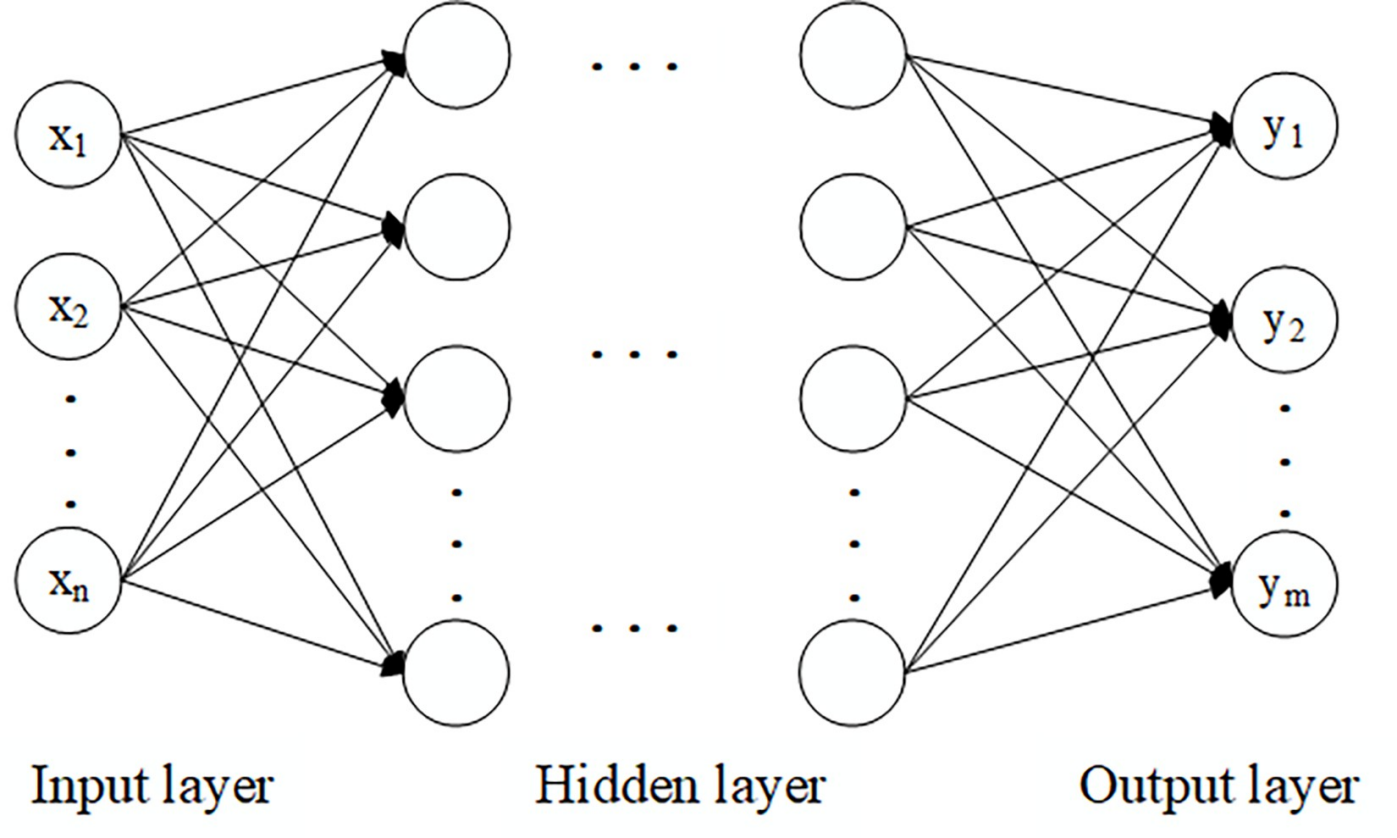
Structure of MLP.

MLP is a widely used neural network model. Each MLP has 3 layers, ① the input layer, ② the hidden layer, and ③ the output layer [24]. The connections between layers are fully connected, with no connections between different layers [25]. The structure was shown in Fig 1.

The data vectors were input into the input layer and passed to the first hidden layer. The output of the hidden unit j for the first hidden layer is

$$a_{j}=Sigmoid(\sum_{i=1}^{n}W_{ij}\cdot x_{i}+b_{j}),$$ (5)

where *a*_*j*_ is the output of the first hidden unit. *W*_*ij*_ is the weight from the input layer to the hidden layer. *x*_*i*_ is the input of the input layer. *b*_*j*_ is the bias of the hidden layer. For the L-th hidden layer, the output of the hidden unit j is

$$a_{p}=Sigmoid(\sum_{i=1}^{p}W_{jp}\cdot a_{j}+b_{p}),$$ (6)

where *a*_*p*_ is the output of the L-th hidden unit. p is the number of neurons in the (L-1)-th hidden layer. *W*_*jp*_ is the weight from the (L-1)-th to the L-th hidden layer. *a*_*j*_ is the input of the L-th hidden layer. *b*_*p*_ is the bias of the L-th hidden layer. The output of the last hidden layer is then passed to the output layer. The input and output of the output unit k for the output layer is

$$y_{k}=ReLU(\sum_{j=1}^{m}W_{pk}\cdot a_{p}+b_{k}),$$ (7)

where *y*_*k*_ is the output of the output unit. *W*_*pk*_ is the weight from the hidden layer to the output layer. *a*_*p*_ is the output of the hidden layer. *b*_*k*_ is the bias of the output layer.

The cross-entropy loss function could be used in MLP model to measure the difference between the output and the labels. The weights W and biases b are updated using the gradient descent algorithm. The cross-entropy loss function could be defined

$$L(y,\hat{y})=-\frac{1}{N}\sum_{i=1}^{N}\sum_{c=1}^{M}y_{i,c}\log\left({\hat{y}}_{i,c}\right),$$ (8)

where *y* is the true probability distribution. *y*_*i*,*c*_ is the indicator variable (0 or 1), which indicates that the i-th sample belongs to c-th category. $\hat{y}$ is the output probability distribution of the model. ${\hat{y}}_{i,c}$ is the probability that the i-th sample belongs to c-th category predicted by the model. N is the number of samples. M is the number of categories.

### 2.3 Attention mechanism

**Fig 2:**
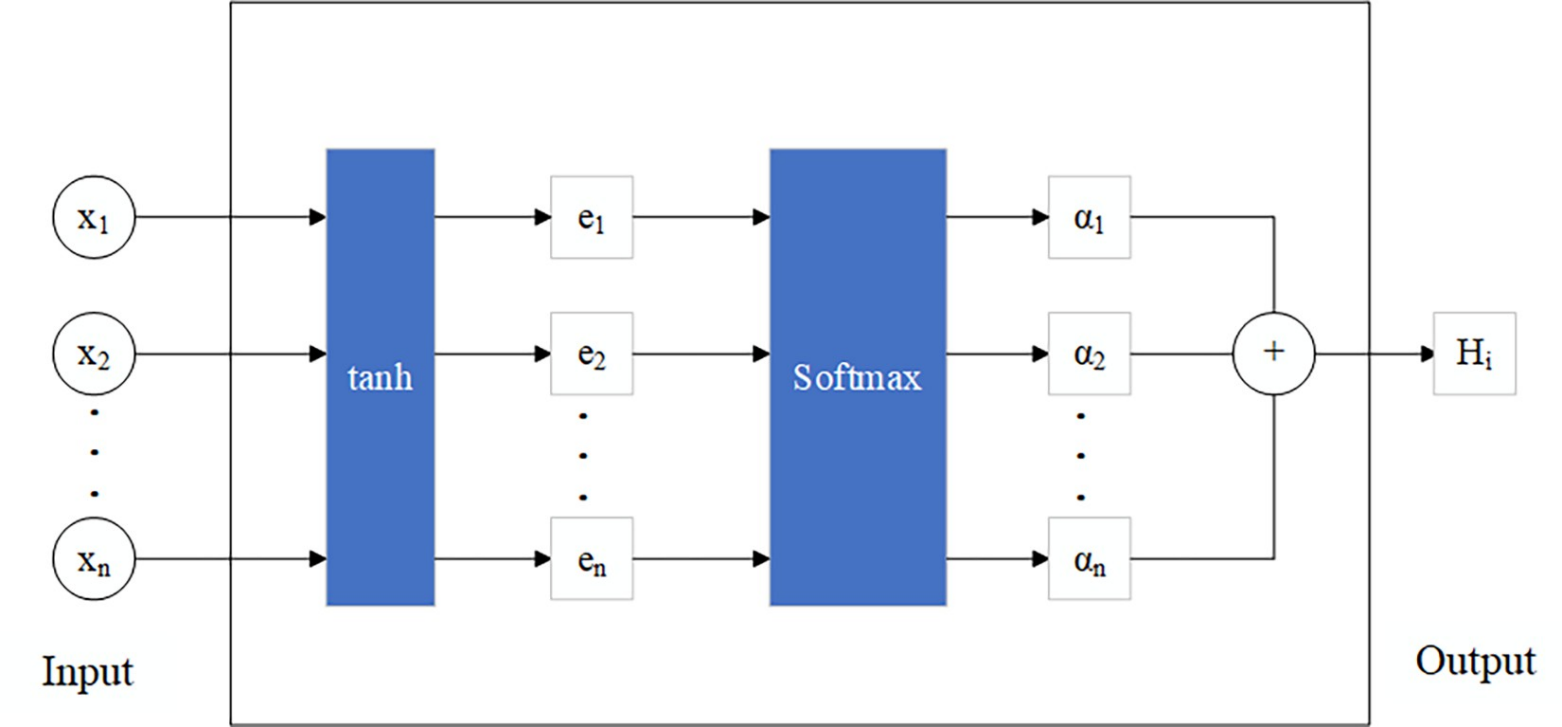
Diagram of attention mechanism.

AM focus on the significant components of the input data by assigning weights, so that the robustness and accuracy could be improved [26,27]. The diagram of AM was shown in Fig 2.

The definition of weight coefficients in AM is

$$\{\begin{array}{c} e_{n}=u\;\tanh\left(w*X+b\right)\\ \alpha_{n}=\mathrm{Softmax}\left(e_{n}\right)=\frac{\exp\left(e_{n}\right)}{\sum_{j=1}^{t}e_{n}} \end{array},$$ (9)

where *u* and *w* are the different weights, *b* is the bias vector, *X* is the input of the AM, *α*_*n*_ is the different weight coefficients.

The M-A model is a neural network model that combines the multi-layer perceptron (MLP) and attention mechanism. In a typical MLP model, the input layer and the hidden layer are connected. The model has output after the process of multiple hidden layers. In the M-A model, the input is processed by an attention mechanism module. The output of the attention mechanism is connected to the hidden layer. In this way, dominated features could be enhanced, and the non- dominated features would be weakened.

### 2.4 Dual-channel MLP attention model

**Fig 3:**
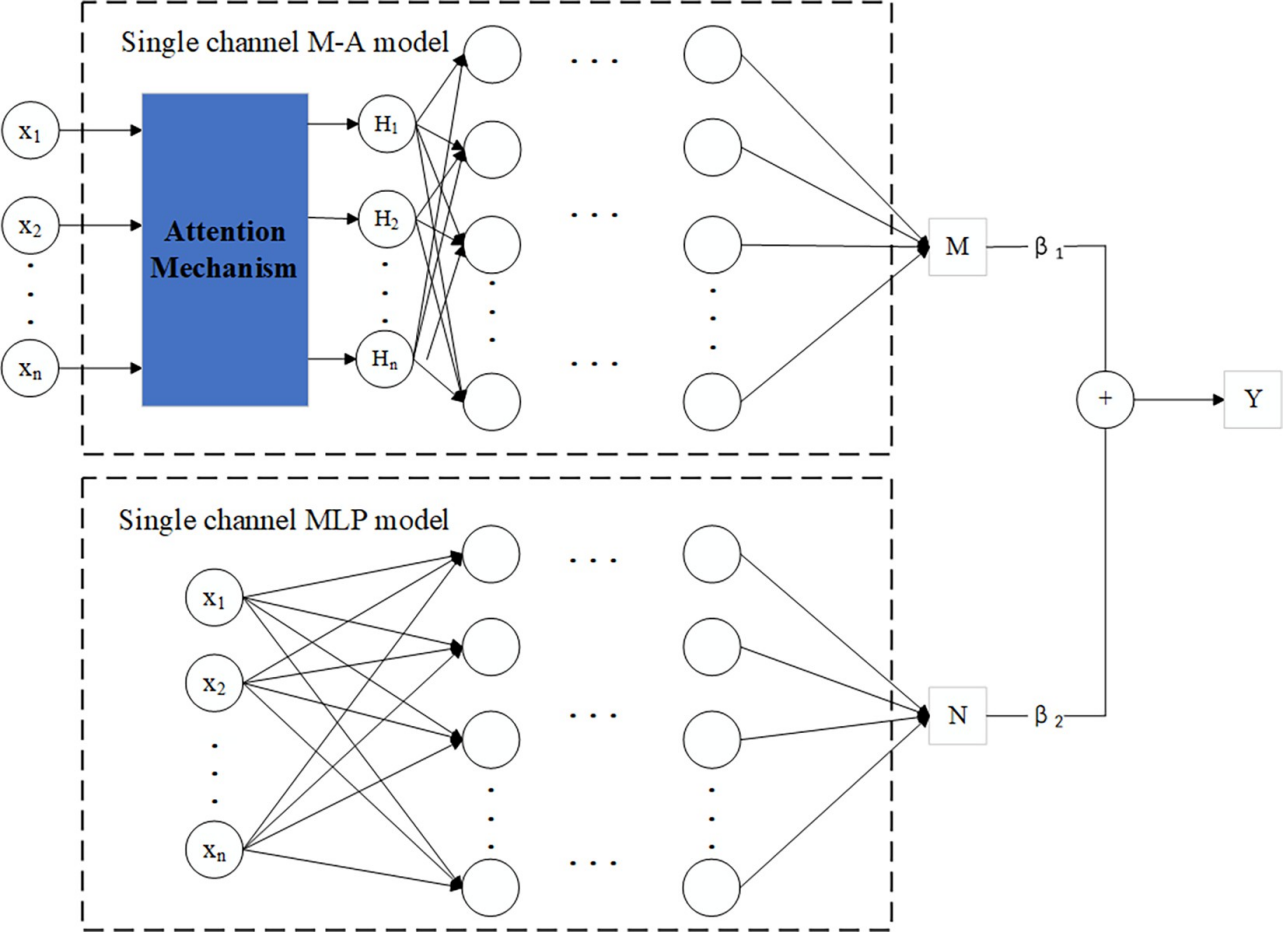
Dual-channel MLP-Attention model.

The structure of the dual-channel MLP-Attention model proposed in this paper is shown in Fig 3. There are two channels. One is a combination of MLP and AM. The input layer of the MLP was optimized by AM. The other channel is MLP. The final output is the weighted result of the two channels. The output of the single-channel M-A model is

$$M=ReLU(X_{att}w_{h1}+b_{h1}),$$ (10)

$$X_{att}=X⊙\alpha ,$$ (11)

The output of the single-channel MLP model is

$$N=ReLU(Xw_{h2}+b_{h2}),$$ (12)

The weighted output Y could be calculated as

$$Y=\beta_{1}\cdot M+\beta_{2}\cdot N,$$ (13)

where *w*_*h*1_ and *w*_*h*2_ are weight matrices. *b*_*h*1_ and *b*_*h*2_ are bias vectors. β_1_ and β_2_ are weight coefficients.

### 2.5 GWO optimization

**Fig 4:**
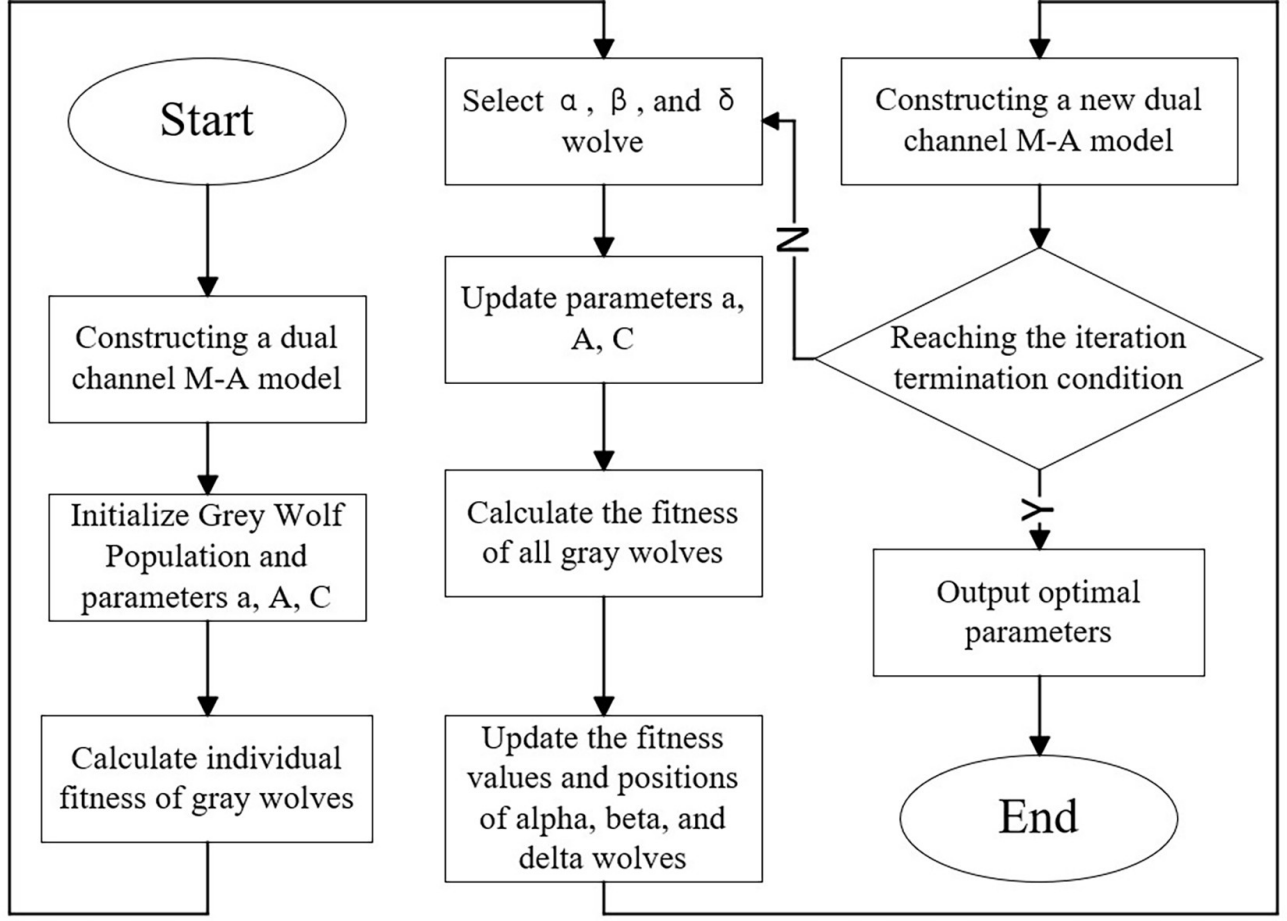
Process of GWO optimization.

The optimization process of the number and nodes of hidden layers of the dual-channel M-A model using GWO was shown in Fig 4.

1. Construct the dual-channel M-A model. The channel 1 is MLP, channel 2 is MLP-Attention.
2. Update the parameters of dual channel M-A model using GWO. Construct a new dual-channel M-A model and train the network.
3. Determine whether the iteration stop condition is satisfied. If satisfied, the optimal parameters would be output.

### 2.6 Faults identification

**Fig 5:**
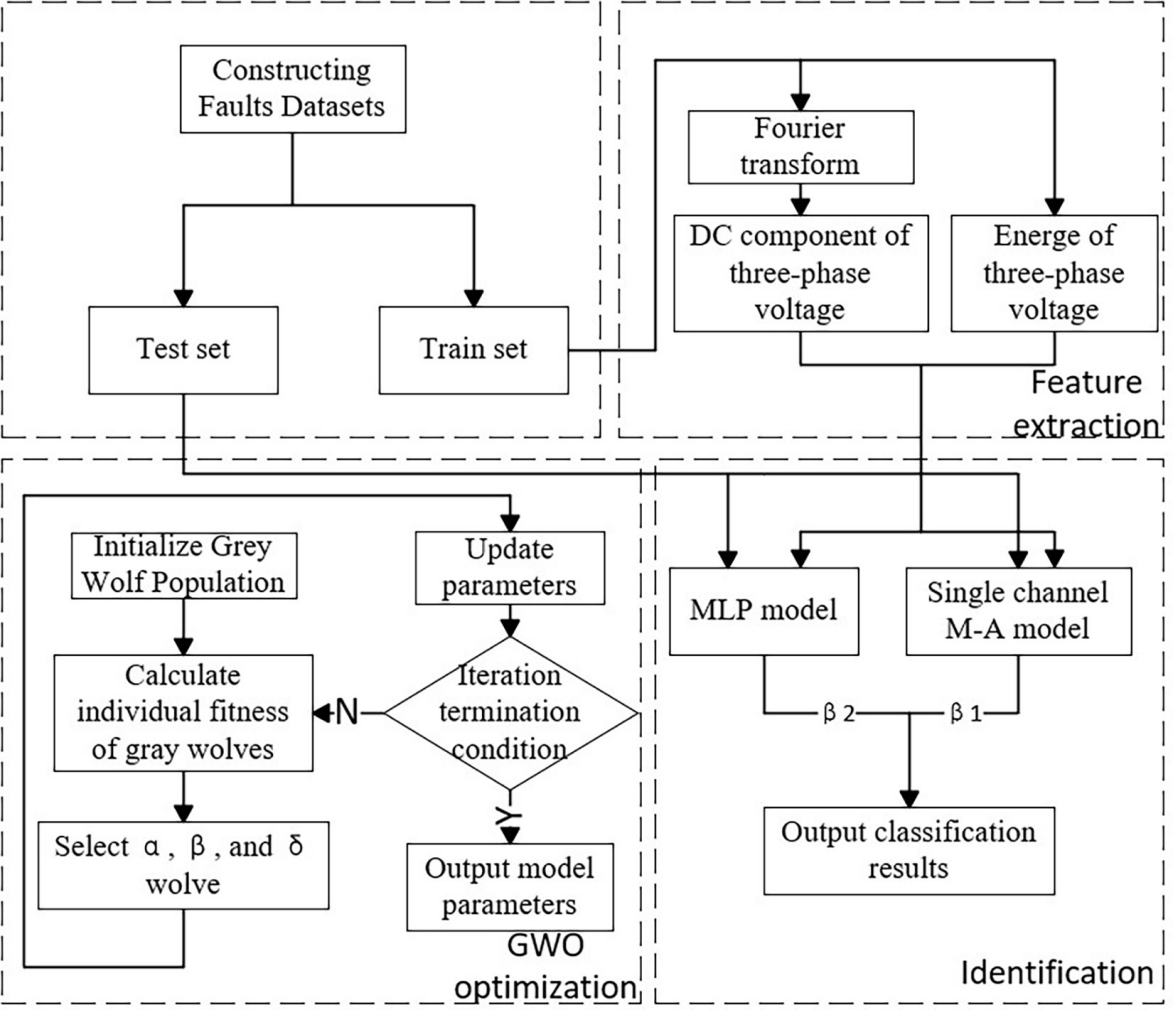
Diagram of faults identification.

The diagram of the fault identification using GWO-optimized Dual-channel M-A model was shown in Fig 5. First, the multi- features were extracted. The three-phase A, B, and C voltage signals were transformed using Fourier method, and the DC components were used as feature 1–3. Then, the energy of the three-phase A, B, and C voltage signals were taken as feature 4–6. All data were randomly divided into training set and test set in a ratio of 8:2. Further, the parameters of the Dual-channel M-A model was optimized by the GWO algorithm to identify the faults.

## 3 Experiment

### 3.1 Simulated system

The data used in the experiment is obtained from the simulation of Digital Dynamic Real-Time Simulator (DDRTS). This system could simulate the operation of substations, including bus faults, line faults, transformer faults, etc.

**Fig 6:**
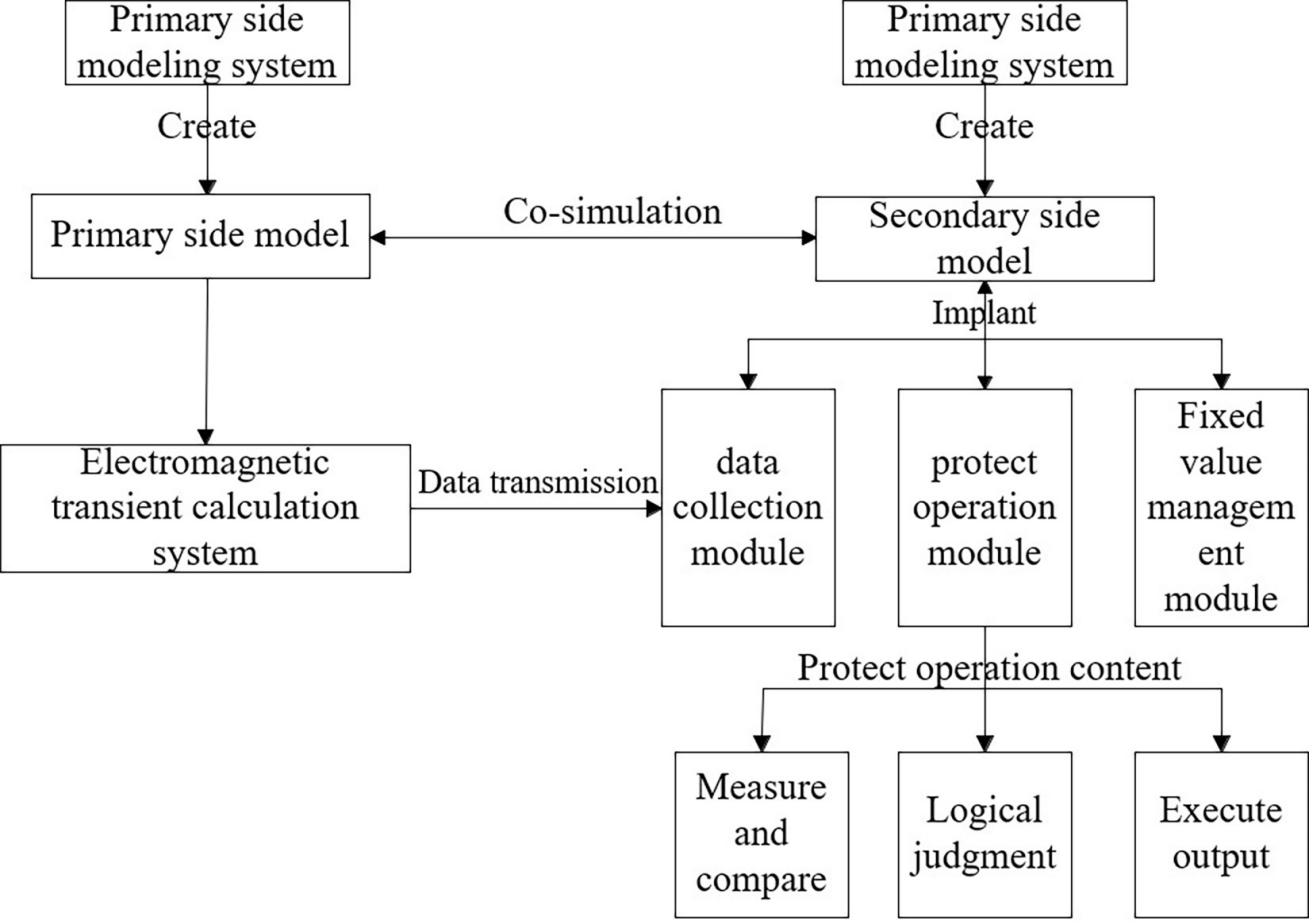
Data flow of virtual digital protection simulation.

The virtual secondary system runs on a graphical simulation platform. The calculated data is obtained from the DDRTS interface. The response and actions of protective devices in actual substation operations can be simulated. The simulation results of the virtual protection devices could be displayed through a visualization interface. The flow of virtual digital protection simulation data was shown in Fig 6.

### 3.2 Data set

There are a total of 1500 transformer fault data. The samples are divided into 10 classes, phase A ground fault, phase B ground fault, phase C ground fault, AB phase-to-phase fault, BC phase-to-phase fault, CA phase-to-phase fault, AB ground fault, BC ground fault, CA ground fault, and ABC ground fault. 1200 samples were used for training, and the remaining 300 samples are used for testing. The sample group was shown in Table 1.

**Table 1:** Sample group.

Fault type	Training samples	Test samples	Fault type	Training samples	Test samples
AN	120	30	CA	120	30
BN	120	30	ABN	120	30
CN	120	30	BCN	120	30
AB	120	30	CAN	120	30
BC	120	30	ABCN	120	30

## 4 Results

### 4.1 Fault coordinate recording

**Fig 7:**
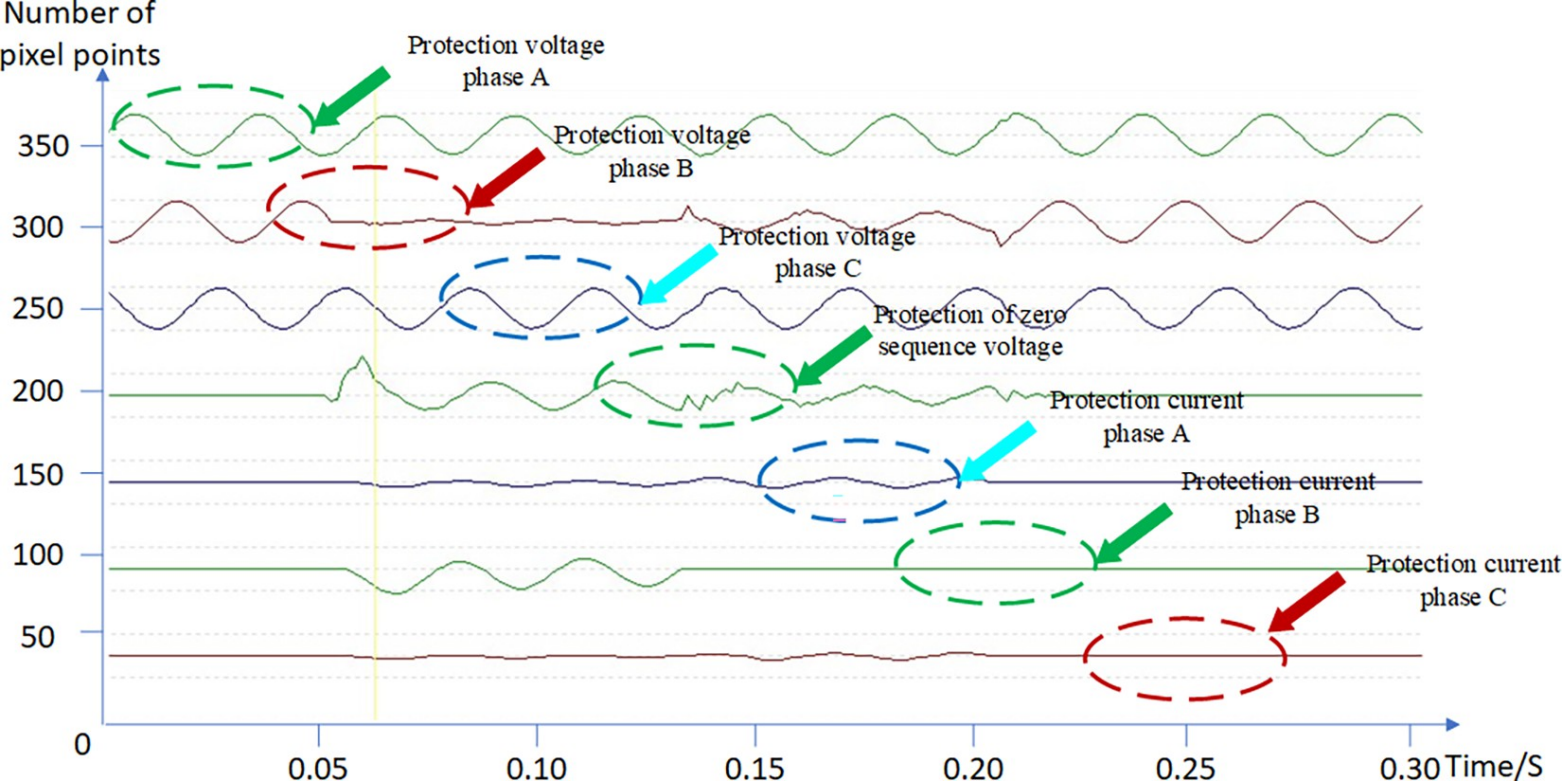
Fault recording signal.

Take the phase B ground fault as an example, the coordinate records (Phase A, B, C protection voltages, protection zero-sequence voltage, and phase A, B, C protection currents) were taken from fault waveform signal, as shown in Fig 7.

### 4.2 Feature extraction

For the A, B, and C phase voltage fault, the coordinates of 100 sampling points were extracted. There are six features in total. Take the absolute value of the three-phase voltage signal and perform Discrete Fourier Transform(DFT) processing. The DC component are taken as features 1–3. The energy of the three-phase voltage signal was features 4–6. The fault types and partial feature data are shown in Table 2.

**Table 2:** Partial faults features.

Faults	Feature 1/kv	Feature 2/kv	Feature 3/kv	Feature 4/db	Feature 5/db	Feature 6/db
AN	364.84	374.93	374.64	25.86	113.14	110.484
BN	374.92	364.00	375.08	112.76	29.26	109.23
CN	374.92	371.83	367.46	110.31	112.68	24.62
AB	370.76	371.91	373.21	73.04	70.74	112.17
BC	375.71	370.80	269.56	114.15	72.19	67.29
CA	372.00	374.01	369.27	66.93	115.10	66.65
ABN	365.08	368.22	374.83	24.05	30.66	107.78
BCN	372.09	368.80	366.83	105.07	28.68	26.62
CAN	365.35	374.97	367.39	22.72	110.29	27.62
ABCN	369.70	366.16	367.96	40.58	37.58	32.68

### 4.3 Optimization results

The GWO-based optimization algorithm has good convergence performance, so that the great solution could be calculated in a short time. In addition, the GWO algorithm requires fewer parameters to be adjusted, so it is suitable for the fault signal processing.

**Fig 8:**
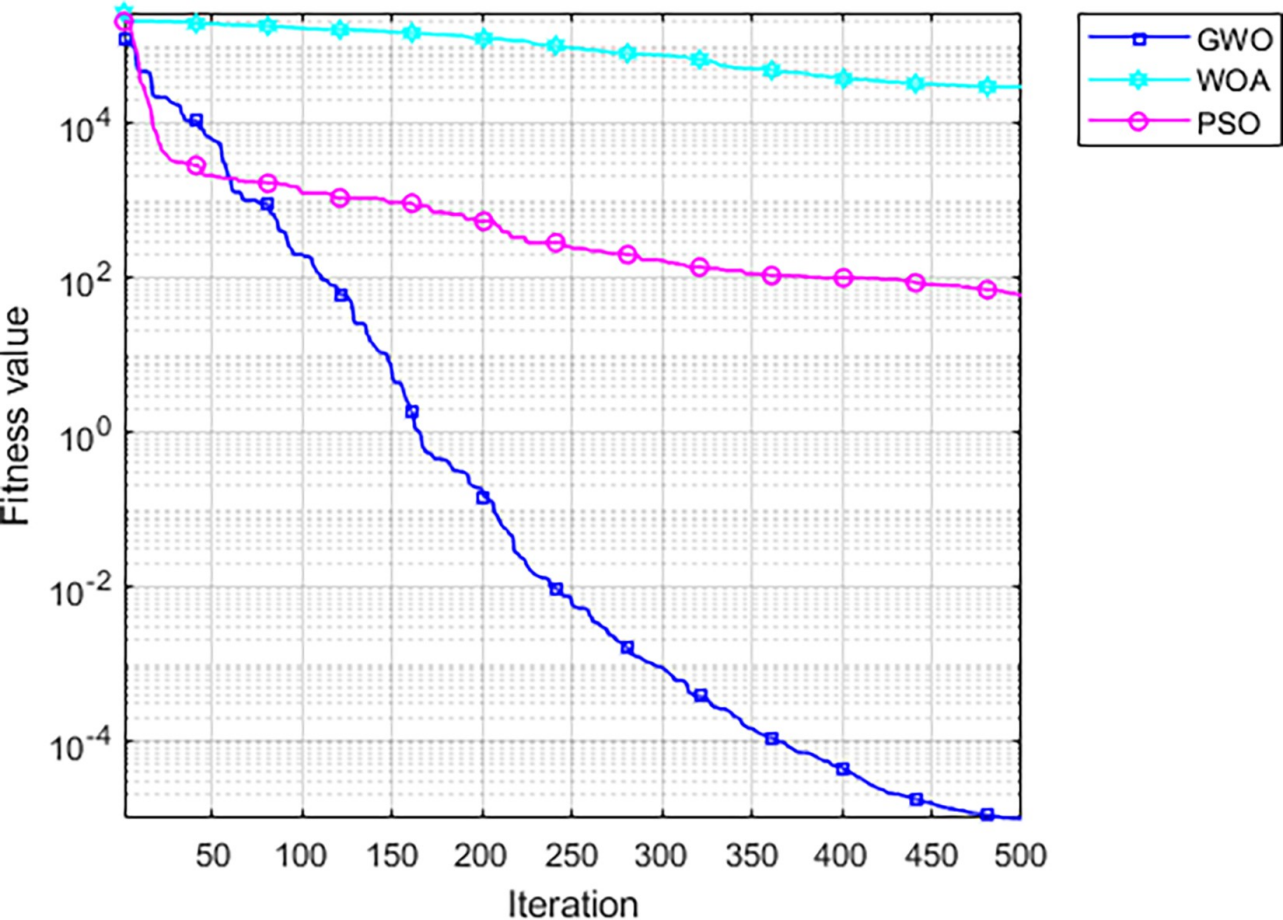
Comparison of optimization algorithms.

Due to population diversity issues, the GWO method has local optima risks. The WOA (Whale Optimization Algorithm) and PSO (Particle Swarm Optimization) method were used to compared with the proposed GWO-based method. The result was shown in Fig 8.

**Fig 9:**
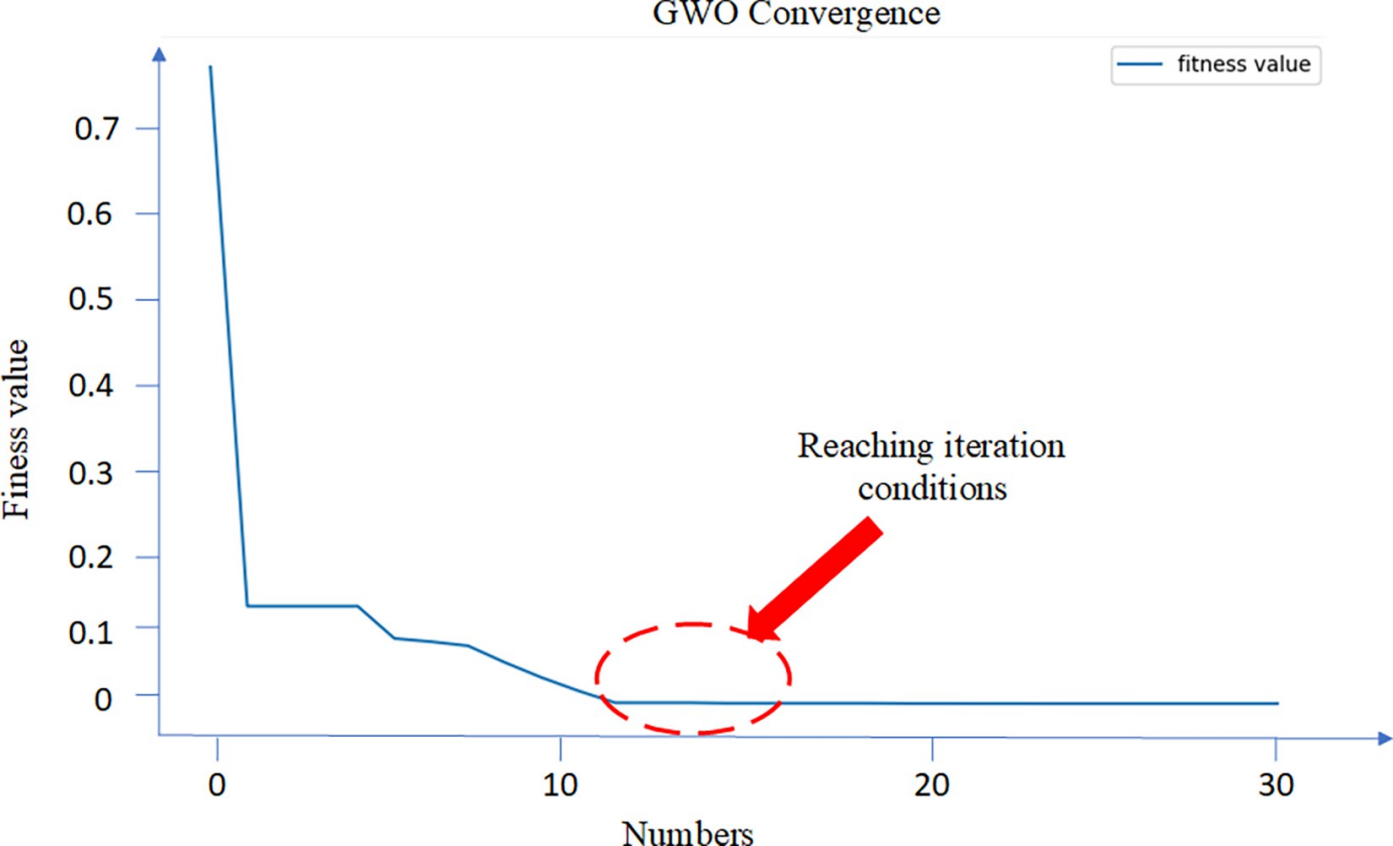
. Fitness curve.

Fig 8 shows that the convergence performance of GWO-based method is significantly better than the other two algorithms. This result shows the advantage of the GWO-based method. The fitness curve was shown in Fig 9. After the 12-th iteration, the fitness value reaches the minimum. The dual-channel M-A model was designed based on the optimization of the number and nodes of hidden layers.

### 4.4 Identification performance

**Fig 10:**
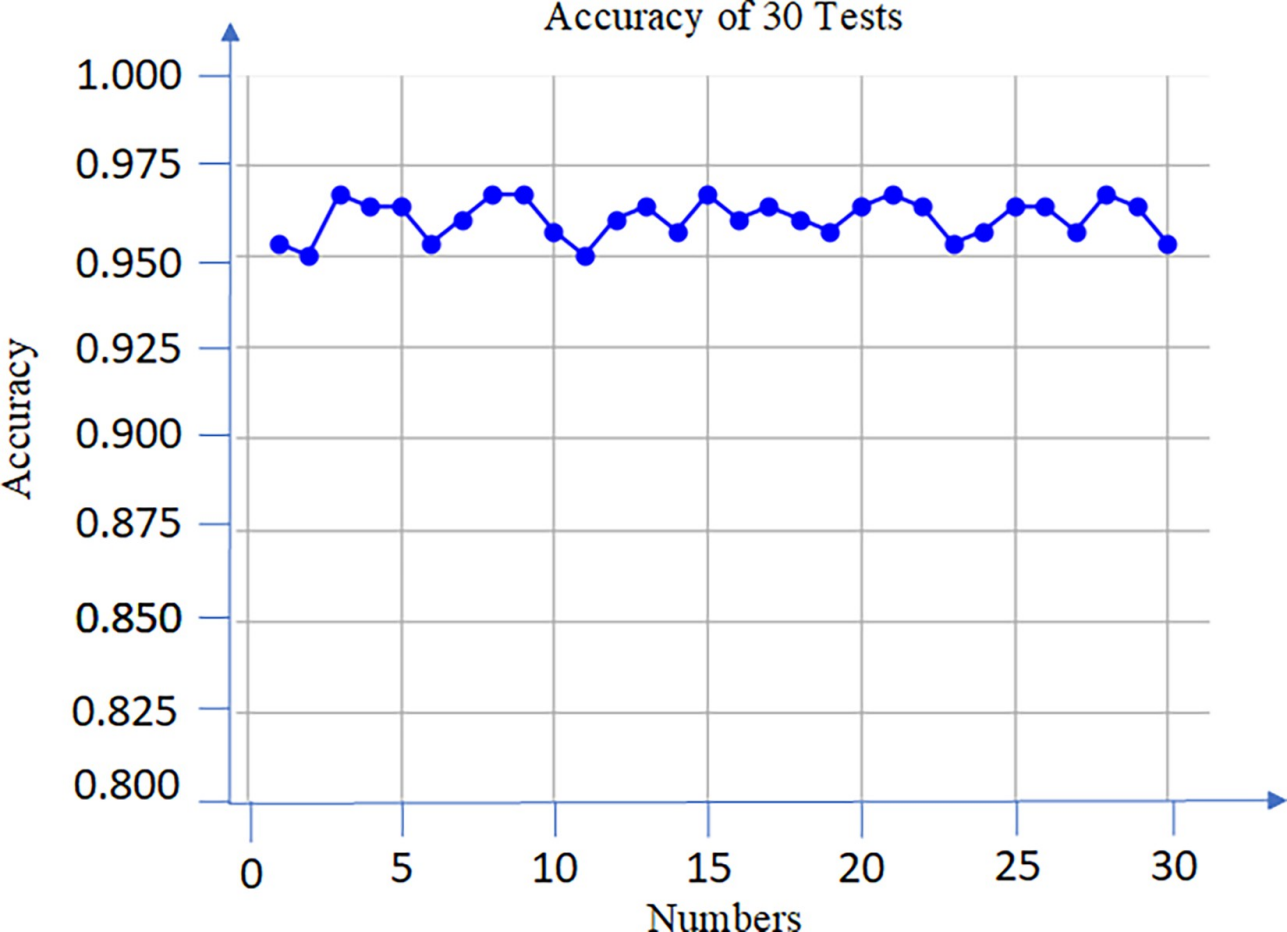
Accuracy curve.

After optimization and training, the accuracy was shown in Fig 10. 30 test experiments were conducted, the accuracy was 95.3% - 96.7%. The experiment shows that the proposed model has a good identification performance for transformer faults.

### 4.5 Ablation study

To validate the performance of the proposed method, an ablation study was conducted. The two channel attention mechanisms were removed in turn, and the identification performance was shown in Table 3. Accuracy rate, Precision (*P*), Recall (*R*), and *F*−*Measure* were used as the metrics to assess the performance of the algorithms. Accuracy rate is the ratio of the number of correctly predicted samples to the total number of samples. This indicator emphasizes the proportion of successful predictions made by the model and can reflect the performance. *F*_*Measure*_ can reflect the shortcomings of Precision and Recall indicator. Thus, the performance of the model on imbalanced datasets can be evaluated. The definitions of *Ar*(Accuracy rate), P, R, as well as *F*_*Measure*_ are

$$Ar=\frac{TP+TN}{TP+TN+FP+FN},$$ (14)

$$P=\frac{TP}{TP+FP},$$ (15)

$$R=\frac{TP}{TP+FN},$$ (16)

$$F_{Measure}=\frac{2\times P\times R}{P+R},$$ (17)

where *TP* is the number of samples that are actually positive and identified as positive. *FN* is the number of samples that are actually positive but identified as negative. *FP* is the number of samples that are actually negative but identified as positive. *TN* is the number of samples that are actually negative and predicted as negative.

**Table 3:** Ablation study.

Algorithm	Accuracy/%	Precision /%	Recall /%	F_1-score_/%
Dual-channel M-A	95.1–96.4	95.3–98	94.9–97.1	94.6–97.5
Remove a channel	91.3–93.7	94–96.7	92–94.8	92.9–95.7
Remove Attention	84.7–88.3	86.7–91.3	83.6–88.1	85.1–89.7

Table 3 shows that after removing one channel and the attention mechanism, the performance deteriorates significantly. This result proves the superiority of the proposed method in the fault diagnosis of transformer.

### 4.6 Algorithm comparison

**Fig 11:**
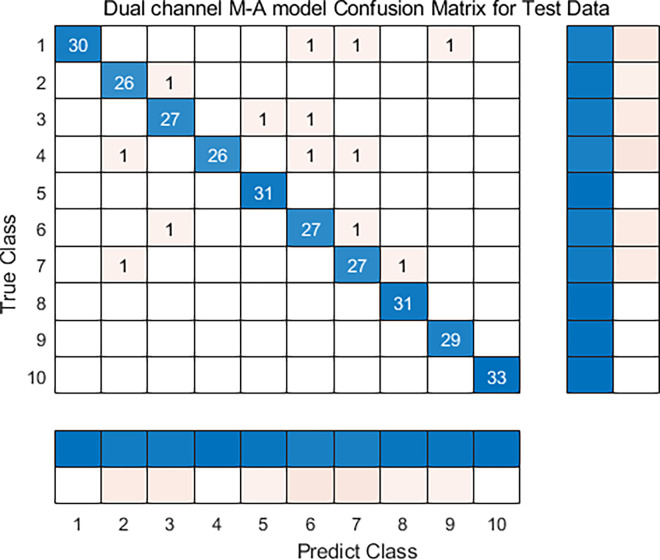
Confusion matrix.

To validate the superiority, the BP (Backpropagation) and SVM (Support Vector Machine) algorithms were used to compared with the proposed method. The confusion matrix of Dual-channel M-A were shown in Fig 11. The comparison results were shown in Table 4.

**Table 4:** Algorithm comparison.

Algorithm	Accuracy/%	Precision /%	Recall /%	F1-score/%
BP	80.6–82.7	81.1–84.3	79.7–83.5	81.3–83.7
SVM	85–87.3	88–90.3	83.5–86.2	85.7–88.2
Dual-channel M-A	95.1–96.4	95.3–98	94.9–97.1	94.6–97.5

Compared with the BP and SVM algorithms, the Dual-channel M-A model has great performance in terms of *Accuracy rate*, Precision (*P*), Recall (*R*), and *F*−*Measure*. This result shows that the proposed method has superior performance in the field of transformer faults identification.

Jin et al. proposed a BP-based transformer fault detection method with the accuracy of 92% [28]. Shan et al. presented an SSA-AdaBoost-SVM method for the fault detection of transformer. The identification accuracy is 91.58% [29]. Andrade Lopes et al. researched an artificial neural network-based transformer fault classification with an accuracy of 85% [30]. Compared with other literature, the proposed method has great performance in the identification accuracy.

## 5 Discussion

The proposed method optimizes the number of hidden layers and hidden nodes by GWO algorithm, so that the generalization ability of the dual channel M-A model could be enhanced. The model can adaptively adjust parameters according to the training scenario. In addition, this research discusses the possibility of optimizing the parameters of identification model using optimization algorithm.

Compared with traditional algorithms, the dual channel M-A model improves the identification accuracy through two channels. The network structure leads to the high computational complexity and long running time. In the future, effective model structures and training algorithms would be explored to reduce the number of parameters and runtime. At the same time, advanced feature fusion strategies would be researched to improve the generalization ability and robustness. The convolutional neural networks (CNNs) in the compressed sensing would be used to reduce the computational complexity. Furthermore, techniques such as Attention Feature Fusion (AFF) can be used to improve the performance of feature fusion. Through the above plan, the performance of transformer fault identification can be further improved.

## 6 Conclusion

Transformer fault may affect the stability of the substation and lead to safety accidents. In this research, a transformer fault identification method based on Dual-channel MLP and attention mechanism was proposed. The main conclusions are

1. By dual channels, the proposed method could learn different features from the dataset simultaneously, which reduces the risk of overfitting. If the performance of one channel degrades, the other channel could provide effective information. Therefore, the model has good robustness.
2. The proposed method could automatically focus on key features by the attention mechanism, thereby improving the accuracy. The accuracy of the proposed method is higher than the traditional MLP method. Thus, it is suitable for the real-time monitoring and fault diagnosis. The experiment shows that the proposed method has good performance in identifying the transformer faults.

## Supporting information

S1 Data Data in the experiment. (ZIP)
